# R5S4TRAIL ameliorates radiation-induced pulmonary fibrosis by alleviating inflammatory responses and promoting apoptosis of fibroblasts

**DOI:** 10.3389/fimmu.2025.1600776

**Published:** 2025-07-31

**Authors:** Yaqin Zhao, Yuanfeng Wei, Wanting Hou, Xianzhou Huang, Qiaoqi Li, Cheng Yi

**Affiliations:** ^1^ Division of Abdominal Tumor Multimodality Treatment, Cancer Center, West China Hospital, Sichuan University, Chengdu, China; ^2^ Department of Radiation Oncology, Cancer Center, West China Hospital, Sichuan University, Chengdu, China; ^3^ Department of Biotherapy, Cancer Center and State Key Laboratory of Biotherapy, West China Hospital, Sichuan University, Chengdu, China

**Keywords:** R5S4TRAIL, radiation-induced pulmonary fibrosis (RIPF), inflammatory, apoptosis, death receptor (DR)

## Abstract

**Background:**

Radiation-induced pulmonary fibrosis (RIPF) is a chronic, fatal and irreversible disease that develops after a consequence of thoracic radiation therapy and few effective treatments have been developed for this condition. Repeated inflammation and excessive accumulation of fibroblasts are features of RIPF. Thus, reducing inflammation and inducing lung fibroblast apoptosis may be an effective strategy for RIPF. Tumor necrosis factor-related apoptosis-inducing ligand (TRAIL), as a natural immunomodulator, can specifically bind to death receptors (DRs) and selectively induce apoptosis in many cells. In our research, we have constructed a novel TRAIL mutant with CPP-like and Smac-like structure (R5S4TRAIL) and aim to explore the role and molecular mechanism of R5S4TRAIL in RIPF.

**Methods:**

Firstly, the RIPF model was established in C57BL/6 mice. Then, the mice were treated with saline (Con group), dexamethasone (Dex group), or R5S4TRAIL (RST group). The remission of RIPF was evaluated by micro-CT, Masson and hematoxylin-eosin (HE) staining. Next, the molecular mechanisms of R5S4TRAIL in RIPF were explored *in vivo* and vitro.

**Results:**

We successfully established the RIPF model and found that R5S4TRAIL treatment could regulate the expression of inflammatory-related cytokines and attenuate the inflammatory response. Meanwhile, R5S4TRAIL treatment could upregulate DR5 expression and induce apoptosis in lung fibroblasts. Briefly, treatment with R5S4TRAIL could alleviate RIPF.

**Conclusions:**

R5S4TRAIL has the potential to ameliorate RIPF by alleviating inflammatory responses and promoting apoptosis of fibroblasts.

## Introduction

Radiation therapy is an important treatment modality for common malignancies such as lung cancer, esophageal cancer, and breast cancer. However, 10% to 30% of patients undergoing thoracic radiation therapy or after treatment may develop radiation-induced lung injury (RILI), which could progress to radiation-induced pulmonary fibrosis (RIPF) (1, 2).The formation of RIPF leads to varying degrees of pulmonary dysfunction, affecting subsequent treatment plans and tumor control, and may even result in patient death (3, 4). Currently, glucocorticosteroids are recommended to reduce inflammation and relieve patients’ symptoms for RIPF. However, the use of high doses of corticosteroids might lead to serious side effects such as necrosis of the femoral head, decreased immunity, and even tumor recurrence and metastasis. No effective treatment has been developed for this condition. Therefore, it is urgent to develop more efficient and targeted drugs to treat RIPF.

Repeated inflammation and excessive accumulation of fibroblasts are features of many fibrotic diseases (5–8). Therefore, reducing inflammation and inducing fibroblast apoptosis may be an effective strategy for treating chronic fibroproliferative diseases (8). Lung fibroblasts and myofibroblasts play major roles in pulmonary fibrosis (6). Studies on idiopathic pulmonary fibrosis (IPF) have shown that fibroblasts exhibit resistance to apoptosis, potentially due to the ineffective activation of the Fas-induced apoptotic pathway (8–10). Thus, inducing apoptosis of lung fibroblasts and reducing inflammation may be a potential strategy to alleviate RIPF.

Tumor necrosis factor-related apoptosis-inducing ligand (TRAIL), a member of the tumor necrosis factor superfamily, is a type transmembrane protein mainly expressed on the surface of leukocytes, including monocytes, macrophages, lymphocytes, and neutrophils (11). When TRAIL binds to death receptors (DRs) through its binding site, it activates the Fas signaling pathway through the death domain (DD) of DR, Fas-associated protein with death domain (FADD), and procaspase 8, forming a death-inducing signaling complex (DISC) and inducing cell apoptosis (12). Current literature reported that TRAIL could reduce inflammation and slow down the progression of some fibrotic diseases (13). In studies on hepatic fibrosis, TRAIL has been shown to reduce liver fibrosis by promoting apoptosis of activated hepatic stellate cells (14–16). In other conditions such as scleroderma and keloids, TRAIL induced apoptosis of myofibroblasts and alleviated the disease (17–19). Therefore, we attempt to explore the role of TRAIL in RIPF. However, wild-type TRAIL has several limitations, such as inadequate induction of apoptosis, the potential development of resistance, which hinder its clinical translation.

In our prior research, we selectively changed five amino acids (VRERG) of the N-terminal in soluble fragments (114–281aa) of the TRAIL protein to form a CPP-like amino acid sequence (RRRRR). Then, we changed the amino acid sequence (PQRV) after the CPP sequence into Smac-like amino acid sequence (AVPI) and developed a mutated form of TRAIL, known as R5S4TRAIL. It exhibited a stronger capability to induce apoptosis than TRAIL (20). Specifically, it could efficiently induce exogenous apoptosis signals through a polyarginine transmembrane peptide-like structure and relieve the inhibitory effect of X-linked inhibitor of apoptosis (XIAP) on endogenous apoptotic pathways by mutating the Smac activity tetrapeptide, thereby combining the intrinsic and extrinsic apoptotic pathways to induce apoptosis effectively and accurately (20–25).

**Scheme 1:**
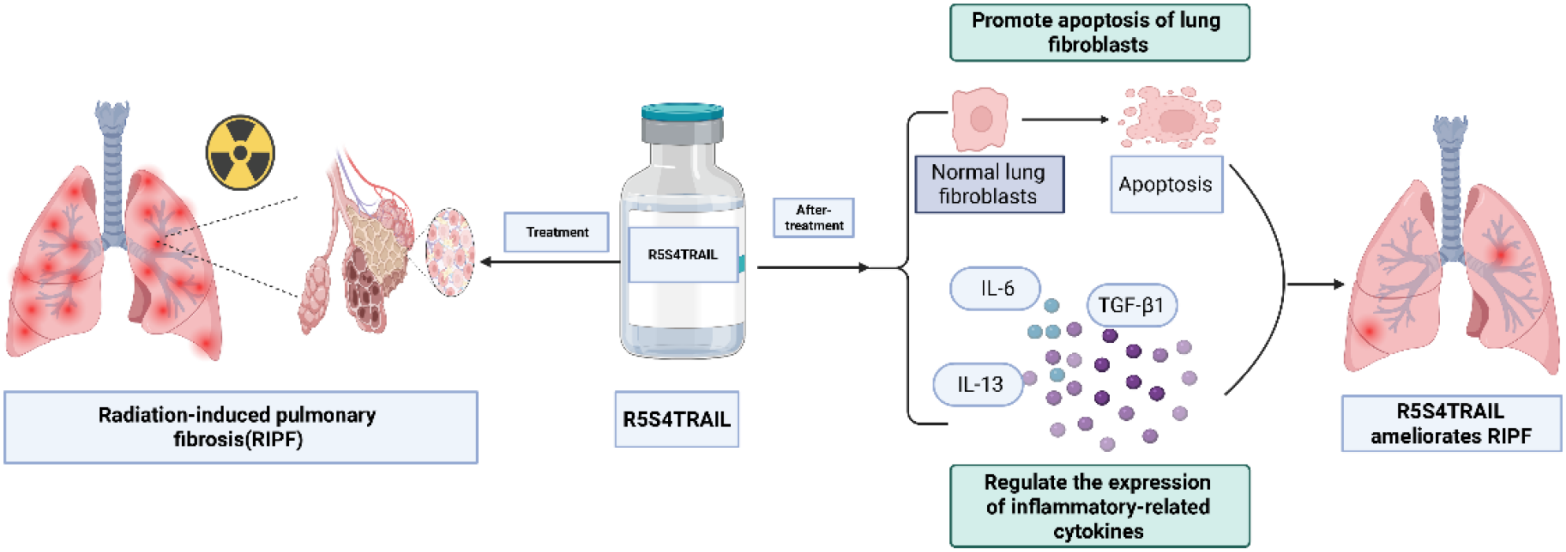
R5S4TRAIL ameliorates radiation-induced pulmonary fibrosis by alleviating inflammatory responses and promoting apoptosis of fibroblasts. Created in https://BioRender.com.

In this study, we attempted to investigate the role and molecular mechanism of R5S4TRAIL in RIPF. Firstly, we established a RIPF mouse model. Then, the mice were treated with saline (Con group), dexamethasone (Dex group), or R5S4TRAIL (RST group). The severity of RIPF was evaluated by micro-CT, Masson, and hematoxylin-eosin (HE) staining. Next, in order to explore the molecular mechanism of R5S4TRAIL in RIPF, the expression of α-SMA and death receptor (DR) in different groups was detected. Subsequently, the expression level of inflammatory indicators (IL-6, TNF-α, TGF-β1, IL-13) was also examined. Moreover, we explored the possible mechanism of R5S4TRAIL in RIPF *in vitro*. Normal human lung fibroblast cells (MRC-5) were irradiated and then treated with dexamethasone (Dex group) or R5S4TRAIL (RST group). Furthermore, proliferation and apoptosis experiments were conducted. Our results indicated that R5S4TRAIL might regulate the expression of inflammatory-related cytokines, attenuate the inflammatory response, and thereby reduce inflammation to ameliorate RIPF. Moreover, R5S4TRAIL treatment could upregulate DR expression and induce apoptosis in lung fibroblasts and then alleviate RIPF. This study aims to provide insights into R5S4TRAIL for the treatment of RIPF (**Scheme 1**).

## Materials and methods

### Cells and animals

The normal human lung fibroblast cell line MRC-5 was cultured in DMEM medium containing 10% fetal bovine serum and incubated at 37°C in a humidified atmosphere of 5% CO_2_. Female C57BL/6 mice (6-week-old, weight 20 ± 2 g) were obtained from Huafukang, Ltd. (Beijing, China). Mice were housed in a pathogen-free room with a controlled environment under a 12 h light–dark cycle, with free access to food and water. This study was approved by the Animal Ethics Committee of West China Hospital, Sichuan University (Project number: 202159A), and all animal procedures were performed in accordance with institutional and national guidelines. Antibodies used in the experiments were purchased from Abcam Inc.

### Establishment and intervention plan of the mouse RIPF model

Anesthesia was delivered via intraperitoneal injection of 1% sodium pentobarbital at a dose of 50 mg/kg. During irradiation, custom lead shielding was used to protect non-target organs, with only bilateral lungs exposed to ensure precise targeting of lung tissue. Specifically, after mice were anesthetized, they were fixed on the mold in the supine position; the head, neck, abdomen, and limbs were covered by a lead block, and only the chest was exposed. Then the bilateral lungs were irradiated by a linear accelerator with a single anteroposterior field using X-rays, with the following parameters: the source-to-skin distance is 100 cm, energy is 6 MV, dose rate is 1.37 Gy/min, and the irradiation field is 3.0×2.0 cm, the prescribed dose is 15 Gy/1f. After 16 weeks of post-irradiation, the degree of RIPF was evaluated by micro-CT and HE staining. Then, mice with equivalent fibrosis levels were randomly divided into three groups. Group 1 served as control group and received intervention with physiological saline, denoted as Con group (n=10); Group 2 served as positive control group and received intervention with dexamethasone sodium phosphate injection at a dose of 2 mg/kg per mouse, denoted as Dex group (n=10); Group 3 served as treatment group and received intervention with R5S4TRAIL injection at a dose of 45 mg/kg per mouse, denoted as RST group (n=10). All three groups were given a volume of 100 μL/mouse, treatments were administered via intraperitoneal injection every other day for three weeks. Micro-CT imaging was performed once a week. Euthanasia was performed at the end of the experiment by intraperitoneal injection of an adequate dose of sodium pentobarbital and their lungs were dissected and weighed. Tissue samples were collected, fixed, and prepared for subsequent analysis.

### H&E and masson staining

After dehydration of the mouse lung tissues, they were sequentially stained with hematoxylin and eosin (HE). Referring to published studies (26, 27), two researchers who were blinded to the treatment groups independently scored the inflammatory reactions under an Olympus microscope. Five random fields were selected, taking into consideration the level of inflammatory cell infiltration in alveolar spaces, thickening of alveolar walls, and the proportion of interstitial edema in lung tissues. The severity of inflammatory lesions in lung tissues was scored according to the following criteria: ≤10% of lung tissue with inflammation was graded 0–1 and scored 0 points; 11% to 30% was graded 2–4 and scored 1 point; 31% to 50% was graded 5–7 and scored 2 points; 51% to 70% was graded 8–9 and scored 3 points; >70% was graded 10 and scored 4 points. The average scores of five fields were collected for statistical analysis.

To detect lung fibrosis, two researchers who were blinded to the treatment groups independently observed the positively stained areas using Masson staining under an Olympus microscope. According to the cumulative area of collagen fiber deposition and the degree of destruction of lung tissue structure, the severity was graded from 0 to 8. Grade 0 represented normal lung tissue and was scored as 0; grade 1~2: mild alveolar septal or peribronchiolar fibrous thickening and was scored as 1; grade 3~4: moderate alveolar septal or peribronchiolar fibrous thickening and was scored as 2; grade 5~6: formation of fibrous strips and localized fibrous foci with some degree of lung tissue destruction and was scored as 3; grade 7: formation of large fibrous foci with severe lung tissue destruction and was scored as 4; grade 8: extensive fibrosis of the whole lung and was scored as 5. Five random fields were selected, and the cumulative area of collagen fiber deposition and the degree of tissue structure damage were graded. The average values for each group were collected for statistical analysis.

### Immunohistochemistry staining

The sections were placed in citrate antigen retrieval buffer and then subjected to antigen retrieval in a microwave oven. After natural cooling, the sections were washed three times with PBS on a decolorizing shaker for 5 minutes each time. The sections were placed in 3% hydrogen peroxide at room temperature for 25 minutes in the dark, followed by three washes with PBS on a decolorizing shaker for 5 minutes each to block endogenous peroxidase activity. The sections were then blocked with 3% BSA solution uniformly covering the tissue in the humid chamber at room temperature for 30 minutes. The primary antibody was added to the chamber and incubated overnight at 4°C. Subsequently, the corresponding species-specific HRP-labeled secondary antibody was added to the tissue, and incubation was carried out at room temperature for 50 minutes. The sections were stained with DAB, with the staining time controlled under a microscope; positive staining appeared brownish yellow. The cell nuclei were counterstained with hematoxylin, followed by bluing and rinsing with running water. After dehydration and air drying, the sections were mounted with neutral resin. Three samples were randomly selected from each group for IHC experiments. For quantitative analysis, five images of positively stained cells were counted from three independent sections per group. The number of positively stained cells was normalized to the number of cell nuclei for each lung tissue sample, and the average value was collected for statistical analysis.

### Real-time Fluorescent Quantitative PCR

Total RNA was extracted from mouse lung tissues, and the first-strand cDNA was synthesized using a reverse transcription kit following the manufacturer’s instructions. The PCR reaction system was prepared. Data analysis was performed using the 2^−△△CT^ method, where △Ct = Ct (target gene) – Ct (reference gene), and △△Ct = △Ct (target gene) – △Ct (control group gene). Each experiment was repeated three times.

### Liquid chip detection

Retro-orbital blood collection was performed under anesthesia to obtain serum. Levels of IL-6, TNF-α, TGF-β1, and IL-13 were detected using a liquid chip assay kit according to the manufacturer’s instructions.

### Cell proliferation assay

Cells in the logarithmic growth phase were harvested, and test samples (dexamethasone sodium phosphate injection, R5S4TRAIL) were diluted with their corresponding complete culture medium to initial concentrations of 1000 μg/mL and 100 μg/mL, respectively. A 3-fold serial dilution was performed to generate a total of 10 concentration points. The samples were then added at a volume of 25 μL per well. After incubation and culture, samples were collected for analysis following the protocol of the cell viability test kit. Data was obtained by measurement of absorbance at 450 nm wavelength to calculate the cell inhibition rate.

### Cell apoptosis induction experiment

Logarithmic phase cells were collected, digested with trypsin and centrifuged. The cells were resuspended in fresh medium and counted. Then, 2 mL of cell suspension was added to a 6-well plate, with a cell seeding density of 2 × 10^5^ cells/mL. The plate was placed in a 37°C, 5% CO2, and 100% relative humidity incubator for 24 h. After irradiation for 2 h, the corresponding drugs (Dex 100 μg/mL, R5S4TRAIL 1.0 μg/mL) were added to the cells and incubated for another 24 h. The supernatant was collected from the culture plate to prepare the cell suspension. Flow cytometry was performed for cell analysis according to the protocol of cell apoptosis detection kit.

### Statistical analysis

The experimental data were analyzed using GraphPad Prism 7 software. Numerical data were presented as mean ± standard deviation. One-way analysis of variance (ANOVA) was used for multiple group comparisons. The independent t-test was performed for comparisons between two groups. A P value of less than 0.05 was accepted as significant. * Indicates P < 0.05, ** indicates P < 0.01, and *** indicates P < 0.001.

## Results

### R5S4TRAIL ameliorates radiation-induced pulmonary fibrosis *in vivo*

**Figure 1:**
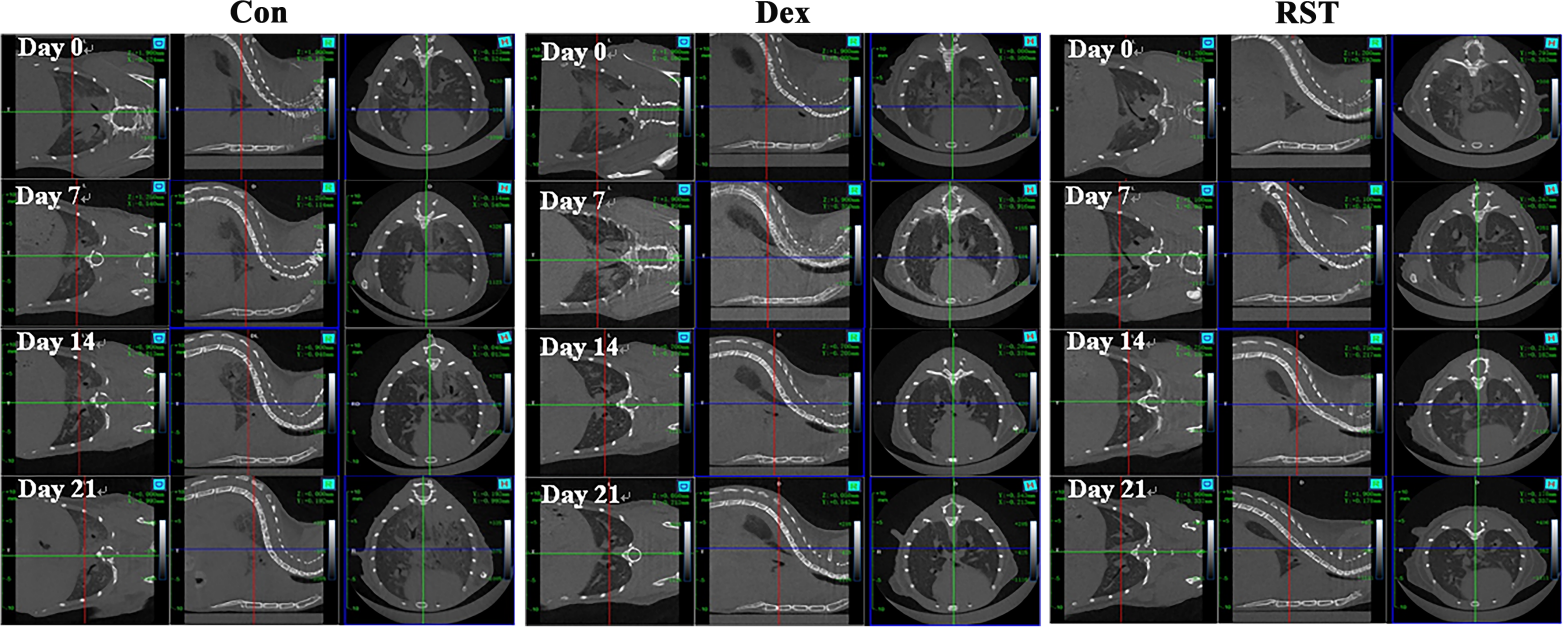
Micro-CT images of lungs for different treatment at different time points.

**Figure 2:**
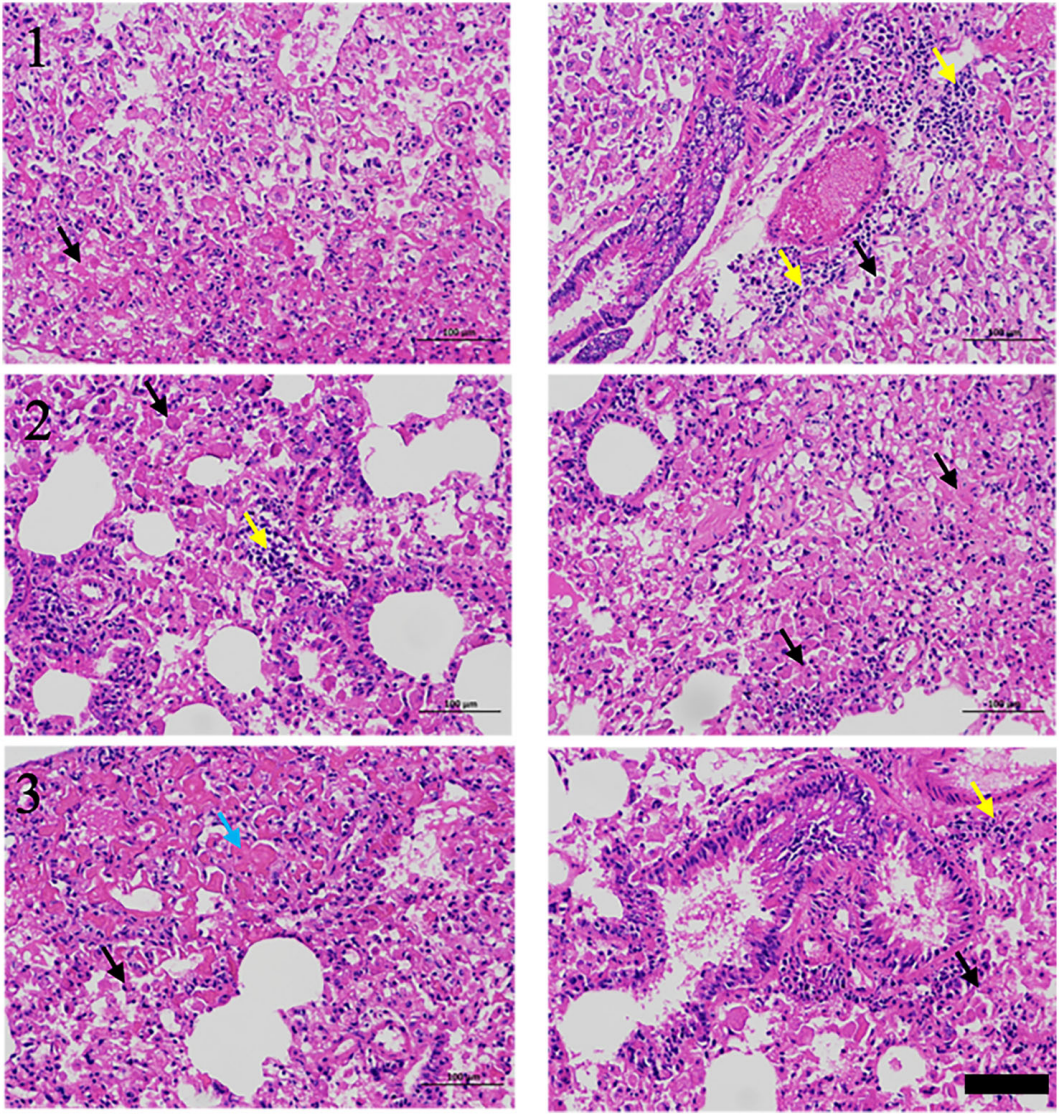
H&E staining of lungs for RIPF model, scale bar=100μm.

RIPF model was established in C57BL/6 mice by single-dose irradiation of 15Gy X-rays to the chest. After 16 weeks of post-irradiation, in order to validate whether the RIPF model was constructed successfully, we performed thoracic micro-CT scanning for each mouse, and the representative images were shown in **Figure 1** (Day 0), which were consistent with the behaviors of pulmonary fibrosis. Subsequently, three mice were randomly selected, and their lung tissues were taken for HE staining. HE staining result presented in **Figure 2**. The results showed that there were many inflammatory cells around the blood vessels in the lung tissue (yellow arrows); the epithelial cells of the alveolar wall were reduced, and fibrin-like exudates and foam cells were observed in the alveolar lumen (black arrows); Moreover, the alveolar lumen structure was blurred and parenchymatous, some alveolar lumens were narrowed, and strong eosinophilic agglutination was observed (blue arrows). The above pathological features were compatible with pulmonary fibrosis.

In summary, micro-CT and pathological results showed that we successfully constructed the RIPF model. Next, we selected mice with similar levels of pulmonary fibrosis by micro-CT results (**Figure 1**) and randomly divided them into 3 groups, which were treated with saline (Con group), dexamethasone (Dex group) or R5S4TRAIL (RST group), respectively. During the treatment for RIPF, we performed regular observations of the lungs in each group using micro-CT. The results showed that the extent of fibrotic lesions gradually increased over time during the treatment process in Con group. However, in the Dex and RST groups, the bilateral ground-glass opacities in the lungs gradually regressed, and the fibrous streaky shadows also reduced gradually (**Figure 1**).

**Figure 3:**
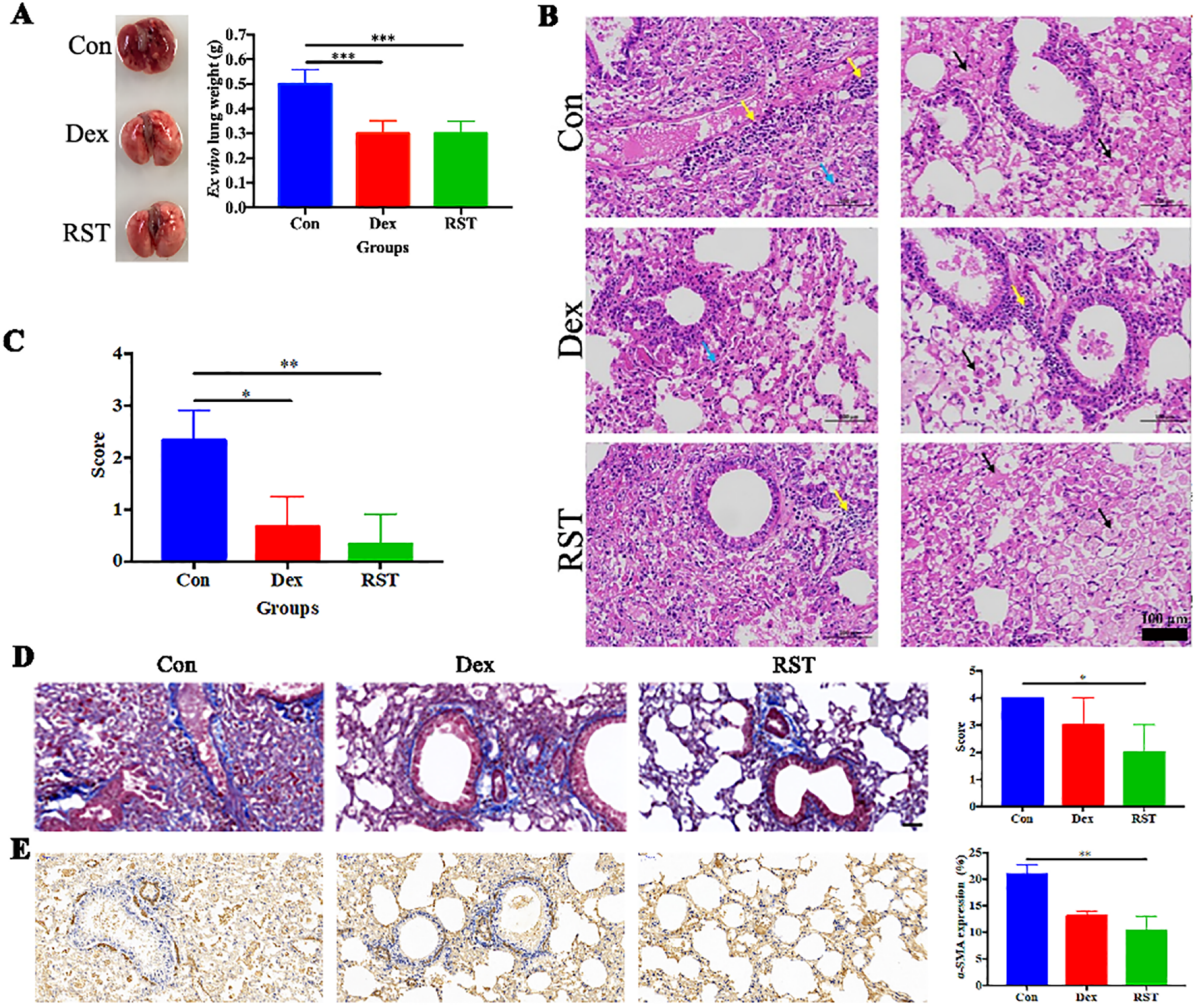
Evaluation of therapeutic efficacy in different treatment groups for RIPF. **(A)** lung images and wet weight in different treatment groups; **(B)** HE staining of lungs after treatment in different groups, yellow arrow: inflammatory cell infiltration; black arrows: fibrinous exudate and foam cells; blue arrow: alveolar stenosis; **(C)** pathological scores of lung lesions with different treatments; **(D)** Masson staining of collagen deposition in different groups; **(E)** IHC staining of α-SMA in different groups. * Indicates P < 0.05, ** indicates P <0.01, and *** indicates P < 0.001.

After the completion of treatment, the lungs of each group were collected for photography, weighing, and histopathological analysis. Visually, the lung tissues of the Con group were dark red in color and widespread erosions were observed, whereas the lung tissues of the Dex and RST groups were pink (**Figure 3A**). The wet weights of the lungs in the Dex group and RST group were lower than in the Con group (P<0.001). There was no significant difference in wet weights of the lungs between the RST group and Dex group (**Figure 3A**). H&E staining showed that the extent of inflammatory lesions in the RST group and the Dex group was lower than in the Con group, and the RST group showed the more obvious remission of pulmonary fibrosis among the three groups (**Figure 3B**). Moreover, we scored the degree of inflammatory lesions in lung tissue in each group. The results were shown in **Figure 3C**, the scores of lung lesions in the Dex and RST groups were lower than in the Con group (P<0.05, P<0.01), the score in the RST group was the lowest. Those results indicated that R5S4TRAIL could ameliorate radiation-induced pulmonary fibrosis.

Although effectiveness is important, safety is also essential. We conducted a preliminary evaluation of the safety of R5S4TRAIL treatment in RIPF. During the experiment, we observed diet, excretion, and behavior every other day. All mice survived and no significant abnormalities were observed. Moreover, we recorded the body weight of mice. The results showed that in the Dex group and RST group, the body weight remained stable. In the Con group, we observed a slight weight loss, possibly related to disease progression (**Supplementary Figure 1**). At the end of the experiment, euthanasia was performed by intraperitoneal injection of adequate sodium pentobarbital and then the main organs (heart, liver, spleen, and kidney) were collected for H&E staining, the results showed that there was no apparent difference between each group (**Supplementary Figure 2**). Moreover, more comprehensive evaluations including blood chemistry, complete blood count, or specific organ function tests, survival and lung function are warranted in future to fully characterize the safety.

### Molecular mechanism of R5S4TRAIL alleviates RIPF *in vivo*

In order to explore the molecular mechanism of R5S4TRAIL in RIPF *in vivo*, relevant experiments were performed. Lung irradiated with high doses of radiation can activate fibroblasts, leading to excessive collagen accumulation in the extracellular matrix. Meanwhile, these activated lung fibroblasts, which proliferate abnormally, express high levels of α-SMA. Masson staining revealed that the blue collagenous staining area in the lung tissues of the Con group was the largest, diffusely distributed in the trachea and alveoli. The Dex group showed less collagen staining, primarily around the trachea and with minimal staining in the alveoli. In the RST group, the staining area was the smallest and mainly concentrated around the trachea (**Figure 3D**). The extent of α-SMA-positive staining in lung tissues of the RST group and Dex group was lower than in the Con group, and the RST group showed the smallest staining area (**Figure 3E**). These results indicated that R5S4TRAIL might reduce collagen deposition and inhibit fibroblast proliferation to ameliorate RIPF.

**Figure 4:**
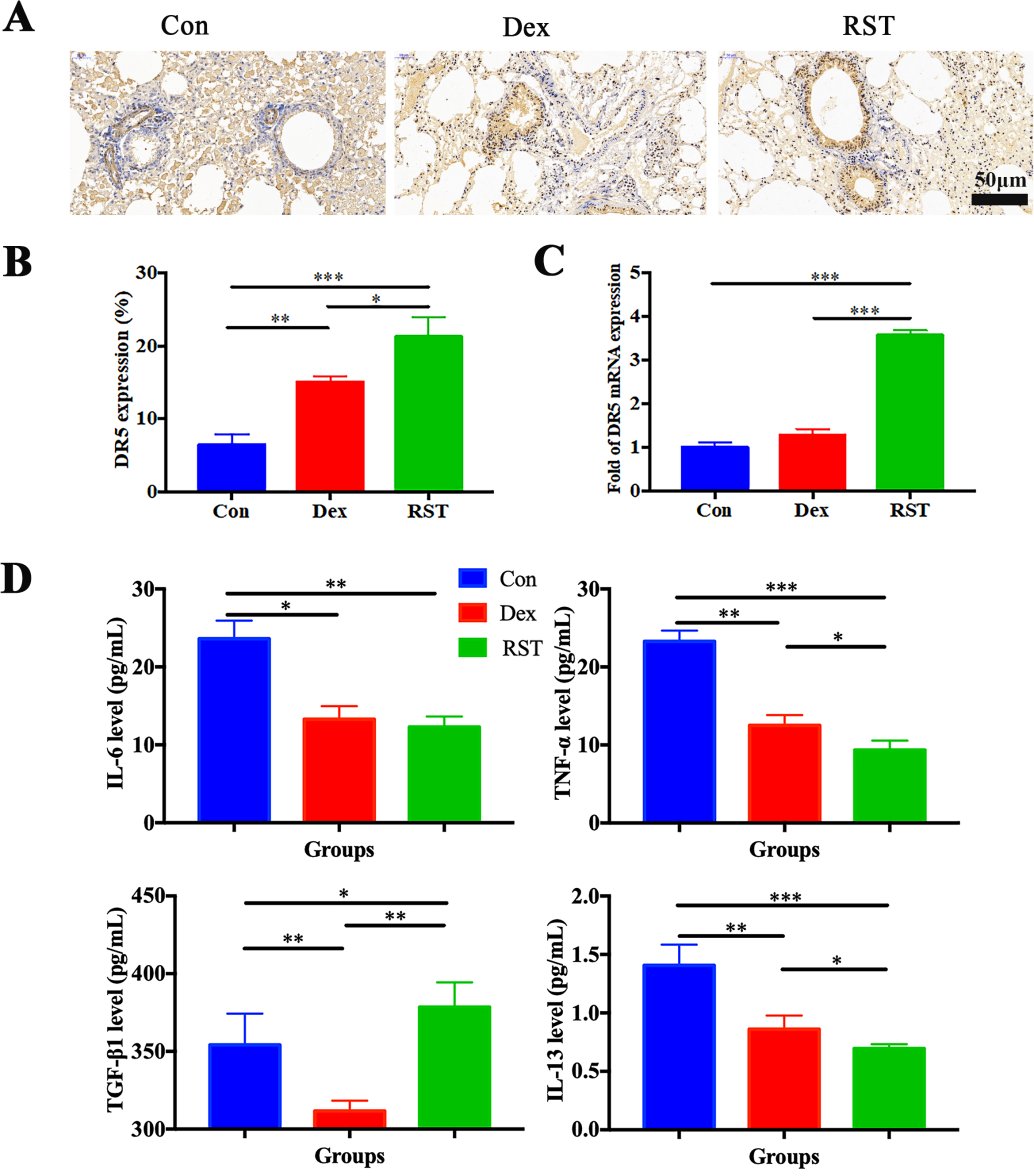
Molecular mechanism of R5S4TRAIL alleviates RIPF *in vivo*. **(A)** IHC staining of DR5 in different groups after treatment; **(B)** Statistical analysis of DR5 IHC staining; **(C)** DR5 mRNA levels in different treatment groups; **(D)** Levels of IL-6, TNF-α, TGF-β1, IL-13 in different treatment groups.

DR5 is the specific target of TRAIL; its high expression facilitates better binding to R5S4TRAIL and thereby promotes apoptosis. IHC analysis showed that the positive staining area and intensity of DR5 in lung tissues in the RST group were significantly higher than in the Con group, and the expression level of DR5 in the Dex group was also increased compared to the Con group (**Figures 4A, B**). PCR results indicated that the mRNA level of DR5 in the RST group was higher than that in the Con group and Dex group (P<0.001) (**Figure 4C**). These results indicated that R5S4TRAIL treatment could upregulate the expression of DR5, thereby promoting the efficacy of R5S4TRAIL in inducing apoptosis of activated lung fibroblasts to alleviate RIPF. Subsequently, to verify whether R5S4TRAIL could reduce inflammation, the mouse serum was collected to detect the expression level of inflammatory indicators (IL-6, TNF-α, TGF-β1, IL-13) by liquid chip technology. The results showed that the expression levels of IL-6, TNF-α and IL-13 in the RST and Dex groups were significantly lower than Con group (P<0.05); RST group was lower than Dex group in the levels of TNF-α and IL-13 (P<0.05); Moreover, the expression level of TGF-β1 was significantly lower in the Dex group than the Con group, whereas it was significantly higher in the RST group than Con group and Dex group (**Figure 4D**). The results indicated that R5S4TRAIL might regulate the expression of inflammatory-related cytokines, attenuating the inflammatory response, thereby reducing inflammation to ameliorate RIPF.

### Molecular mechanism of R5S4TRAIL ameliorates RIPF *in vitro*

**Figure 5:**
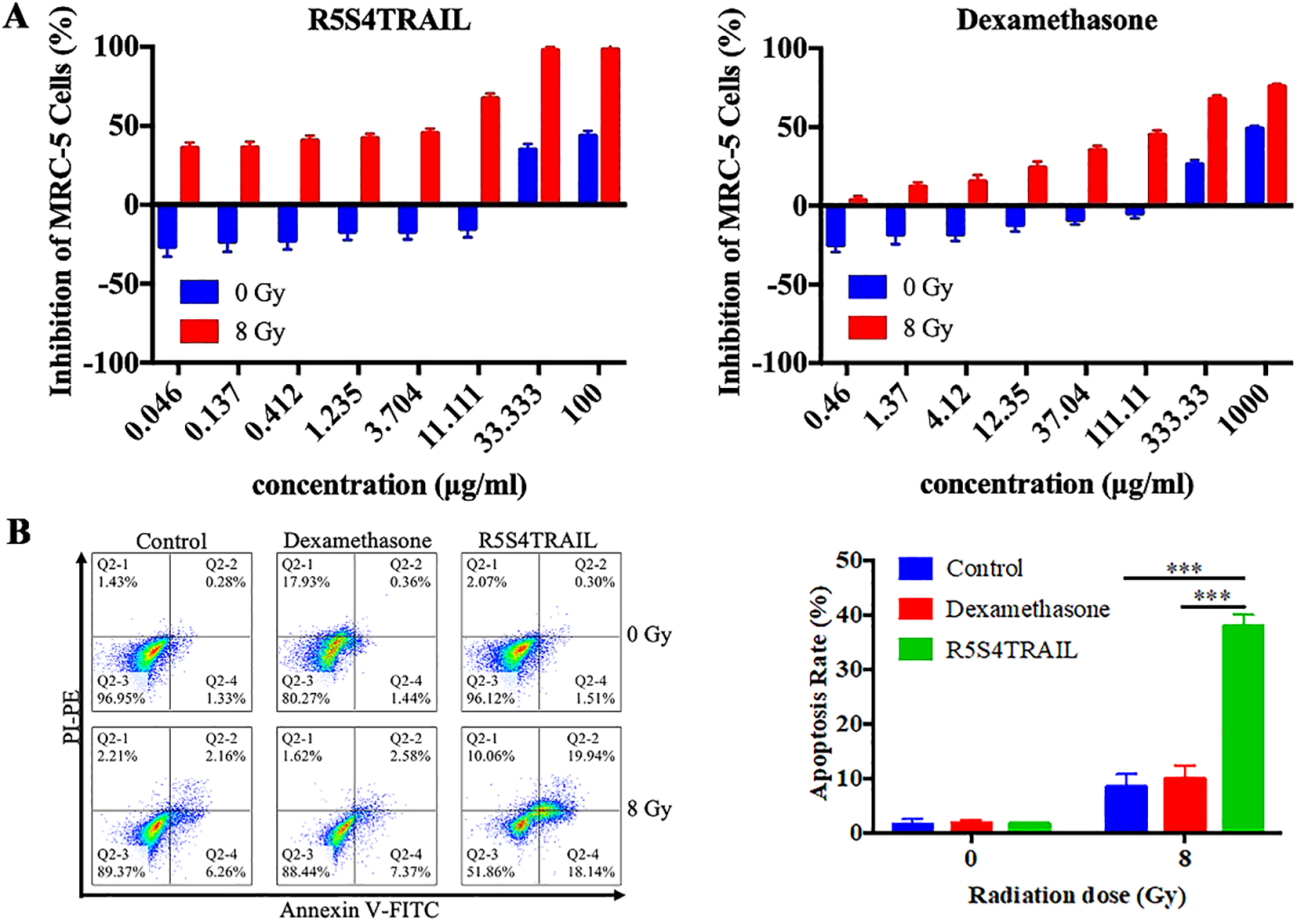
R5S4TRAIL inhibits proliferation and induces apoptosis of irradiated MRC-5 cells. **(A)** Inhibition of MRC-5 cell proliferation by R5S4TRAIL or dexamethasone (Dex) after irradiation with 0 Gy or 8 Gy; **(B)** Apoptosis rate of MRC-5 cells treated with R5S4TRAIL or dexamethasone (Dex) after irradiation with 0 Gy or 8 Gy. *** indicates P < 0.001.

From the above findings, we observed reduced collagen deposition and decreased α-SMA expression in lung tissues following R5S4TRAIL treatment, which may be associated with diminished fibroblasts. To investigate whether R5S4TRAIL has an inhibitory effect on lung fibroblasts after radiotherapy, we selected human embryonic lung fibroblasts MRC-5 for the study of *in vitro*. After irradiation with 8Gy or 0Gy, MRC-5 cells were treated with dexamethasone (Dex) or R5S4TRAIL. The results showed that Dex and R5S4TRAIL had no significant inhibitory effects on MRC-5 proliferation in the absence of irradiation. However, under 8 Gy irradiation, Dex and R5S4TRAIL exhibited strong inhibitory effects on MRC-5 cell proliferation, with R5S4TRAIL showing a more pronounced inhibitory effect than Dex (**Figure 5A**). Next, we performed apoptosis experiments, the upper right and lower right quadrant (Q2–2 and Q2-4) were used for quantification in the flow cytometry analysis. We found that R5S4TRAIL did not increase the apoptosis of non-irradiated MRC-5 cells. However, after receiving 8 Gy irradiation, the proportion of apoptotic MRC-5 cells induced by R5S4TRAIL increased rapidly, significantly higher than in the Dex group and Con group (P<0.001) (**Figure 5B**), it suggested that R5S4TRAIL could increase the apoptotic rate of fibroblasts after radiation. Those above results indicated that R5S4TRAIL could inhibit proliferation and induce apoptosis of MRC-5 cells after irradiation.

## Discussion

Radiation-induced pulmonary fibrosis (RIPF) is a chronic, fatal and irreversible disease that develops as a consequence of thoracic radiation therapy (28, 29). There is no effective treatment that has been developed for this condition. In the present study, we explored the potential role and mechanism of R5S4TRAIL in RIPF. Our results showed that R5S4TRAIL could regulate the expression of inflammatory-related cytokines, reduce inflammatory reactions, and promote apoptosis of fibroblasts through DR targets, thereby alleviating RIPF.

Previous studies revealed that TRAIL could regulate the expression of inflammatory cytokines and induce apoptosis of fibroblasts in fibrotic diseases including hepatic fibrosis, scleroderma, scar hypertrophy, and bleomycin-induced pulmonary fibrosis (15, 17, 30–33). However, its role in RIPF is unclear. Lung fibroblasts and myofibroblasts are the main effector cells in pulmonary fibrosis (34). Moreover, repeated inflammation is also contributed to pulmonary fibrosis (35). Thus, inducing apoptosis of lung fibroblasts and reducing inflammation may be a potential strategy to alleviate RIPF. R5S4TRAIL is a TRAIL mutant we previously developed, which could rapidly bind to DR4/5 on the cell membrane, thereby efficiently and specifically inducing apoptosis. Therefore, R5S4TRAIL might be effective in treating RIPF. In this study, we first constructed radiation-induced pulmonary fibrosis models. To prevent off-target effects on non-fibrotic tissues, custom lead shielding was used to protect non-target organs, with only the bilateral lungs exposed to ensure the precisely targeted to lung tissue during irradiation. In addition, hair, diet, excretion, body weight and behavior were observed during irradiation to access off-target effects and safety. After successful model establishment, the mice were treated with dexamethasone sodium phosphate or R5S4TRAIL. Micro-CT showed that the extent and severity of ground glass opacities in the lungs gradually increased in the control group, indicating disease progression. However, in the Dex and RST groups, the bilateral ground-glass opacities gradually regressed, and the fibrous streaky shadows also gradually reduced. In addition, the efficacy of the RST group was better than the Dex group. These results indicated that R5S4TRAIL could alleviate RIPF.

Moreover, we explored the molecular mechanism of R5S4TRAIL in RIPF. Firstly, we investigated whether R5S4TRAIL could ameliorate RIPF by inducing fibroblast apoptosis. α-SMA is a hallmark protein of activated fibroblasts, which is widely used to detect the number and distribution of fibroblasts (36). Previous studies have shown that TRAIL-related proteins used in liver fibrosis and skin fibrosis were associated with decreased α-SMA expression (37). In our study, the expression of α-SMA in lung tissues was significantly lower in the RST group than in the Con group. The α-SMA positive cells in the RST group were mainly concentrated around the bronchi and sparsely distributed in the alveolar structures. Furthermore, Masson staining revealed that the blue collagenous staining area in lung tissues of the Con group was the largest, with diffuse distribution in the trachea and alveoli. In the RST group, the staining area was the smallest and mainly concentrated around the trachea. These results indicated that R5S4TRAIL treatment might reduce collagen deposition and inhibit fibroblast proliferation. However, in our study, we evaluated the degree of lung fibrosis only by histological analysis. Biochemical assays such as hydroxyproline quantification could further validate our findings. We propose that integrating both approaches in future research would provide a more comprehensive assessment of fibrosis.

Furthermore, the mechanisms underlying the role of R5S4TRAIL deserve further exploration. Several studies have reported that TRAIL could specifically bind to death receptors (DRs) and induce apoptosis in target cells. Our study indicated that R5S4TRAIL treatment could upregulate DR5 levels in RIPF. We postulated that the alleviation of RIPF by R5S4TRAIL might also be related to upregulated DR, which induced caspase cascade reaction, then promoted the cleavage of apoptotic effector caspase-3 and finally induced apoptosis in lung fibroblasts. Previous literature on the anti-fibrotic effects of TRAIL had also shown that its target cells were primarily mesenchymal cells such as stellate cells and fibroblasts; TRAIL could upregulate DR expression, and induced apoptosis (17, 31, 32). To validate whether R5S4TRAIL has an inhibitory effect on lung fibroblasts, we selected human embryonic lung fibroblasts (MRC-5) for *in vitro* studies. The results showed that under 8 Gy irradiation, both Dex and R5S4TRAIL showed strong inhibitory effects on MRC-5 cell proliferation. Moreover, we performed apoptosis experiments. After 8 Gy irradiation, the proportion of apoptotic MRC-5 cells induced by R5S4TRAIL increased rapidly, which was significantly higher than that in the Dex group and Con group. This suggested that R5S4TRAIL could increase the apoptotic rate of fibroblasts. Those above results indicated that R5S4TRAIL could inhibit proliferation and induce apoptosis of MRC-5 cells. In summary, R5S4TRAIL could promote apoptosis of fibroblasts through DR targets, thereby alleviating RIPF.

In addition, the development of RIPF is correlated with repeated inflammation. Moreover, inflammatory factors played an important role in this process. Some studies have reported that radiotherapy could prompt alveolar epithelial cells to release many cytokines, such as TNF-α, IL-6, IL-13, and TGF-β1, which in turn affect the development of pneumonia. In our study, the results showed that the expression level of TGF-β1 was significantly lower in the Dex group than in the Con group, whereas it was significantly higher in the RST group than in the Con group and Dex group. There is conflicting data regarding the role of TGF-β1. Some clinical trials demonstrated that the incidence of radiation pneumonitis (RP) was significantly correlated with plasma TGF-β1 level. The incidence of RP was significantly higher when TGF-β1 levels increased during radiotherapy or failed to normalize after radiotherapy (38). Fleckenstein et al. reported that radiation-induced hypoxia occurred within a few days of radiotherapy and worsens over time in different animal models (39), and that hypoxia could induce upregulation of TGF-β expression (38). Thus, the elevation of TGF-β1 may be related to radiation therapy, although radiotherapy is finished, it failed to normalize after radiotherapy. Moreover, Vladimir V. Yurovsky reported that soluble TRAIL could upregulate the level of TGF-β1 (40). Jong-Sung Park et al. found that TGF-β could upregulate DR expression in fibroblasts through the Smad2/3 signaling pathway, especially more prominently on DR5 (17). The significantly higher TGF-β1 level in the RST group might result from the interaction between TRAIL and TGF-β signaling pathways during therapeutic intervention. In addition, IL-13 also plays an important role in fibrosis (41–43). When IL-13 binds to its receptor, it could activate Janus kinase 2 (JAK2) via the STAT6 signaling pathway, resulting in phosphorylation and nuclear translocation of STAT6. IL-13 transfection along with a type I collagen promoter construct increases promoter activity, while co-transfection with a dominant-negative ERK plasmid reduces promoter activity and collagen secretion. These findings indicate that IL-13 regulates collagen expression via the STAT6 pathway (31, 44). In addition, some studies have reported that fibrosis could be attenuated by regulating the levels of inflammatory immune cytokines, such as IL-13 and others. To validate whether R5S4TRAIL could regulate the expression of inflammatory-related cytokines and thereby reduce inflammation. The expression level of inflammatory indicators (IL-6, TNF-α, TGF-β1, IL-13) was examined. The results showed that the expression levels of IL-6, TNF-α and IL-13 in the RST and Dex groups were significantly lower than in the Con group; additionally, the RST group was lower than the Dex group in the levels of TNF-α and IL-13. Those results indicated that R5S4TRAIL might regulate the expression of inflammatory-related cytokines, attenuate the inflammatory response, and thereby alleviate RIPF.

However, there are still some limitations and challenges. First, biochemical assays such as hydroxyproline quantification, combined with HE and Masson staining, could further validate the degree of lung fibrosis. We propose that integrating these approaches in future research would offer a more comprehensive assessment of fibrosis. Second, we evaluated the safety of R5S4TRAIL only via body weight, and HE staining of main organs. More comprehensive evaluations including blood chemistry, complete blood count, specific organ function tests, survival and lung function are warranted in future studies to fully characterize the safety. Third, we only used the human embryonic lung fibroblasts cell line MRC-5 to investigate the effect of R5S4TRAIL on lung fibroblasts after radiotherapy. Incorporating additional models, such as primary fibroblasts from irradiated mice or human fibrotic tissue, would better reveal the reality of our conclusions. Furthermore, MRC-5 cannot fully capture the complexity of primary cells. Recent studies indicate that the WI-38 cell line is emerging as a compelling alternative, offering reproducibility, phenotypic plasticity, and functional relevance. It could replicate the phenotypic and transcriptomic characteristics of primary lung fibroblasts from patients with idiopathic pulmonary fibrosis (IPF) under various conditions (45, 46). In our subsequent experiments, we will consider replacing MRC-5 cells for experiments. Fourth, adding dose-response and time-course experiments to assess apoptosis and proliferation would better clarify the results and provide value for optimizing the application of R5S4TRAIL in future translational studies. These experiments are planned for our follow-up work. Moreover, we preliminarily selected dexamethasone sodium phosphate as a control. Pirfenidone and nintedanib are clinically recommended for the treatment of idiopathic pulmonary fibrosis. In subsequent study, we will include pirfenidone and nintedanib to further compare the efficacy of R5S4TRAIL in pulmonary fibrosis. Furthermore, R5S4TRAIL as a new pharmaceutical, there are still various challenges in clinical translation. Achieving targeted delivery to the lung parenchyma is crucial for the effective treatment of pulmonary fibrosis. Systemic administration of R5S4TRAIL could potentially encounter issues such as low bioavailability, rapid elimination, and off-target toxicity. Additionally, the physical and chemical properties of the molecule may also impede its penetration into fibrotic tissue and lead to degradation *in vivo*. Moreover, TRAIL-based therapeutics are at risk of triggering immune responses, which may reduce their efficacy or cause inflammatory reactions. Off-target activation of immune cells via Fc-mediated interactions could also result in systemic toxicity. To systematically address these challenges, further animal and clinical trials should be conducted to confirm the safety and efficacy of R5S4TRAIL in pulmonary fibrosis. Finally, RIPF typically develops as a complication of thoracic radiotherapy in cancer patients, where the tumor microenvironment significantly influences inflammation and fibrosis. In our future studies, we would be incorporating a tumor-bearing model to simulate clinical scenarios, thereby providing deeper insights into the interactions between tumors, radiation, and fibrotic remodeling.

## Conclusions

R5S4TRAIL has the potential to ameliorate RIPF by alleviating inflammatory responses and promoting apoptosis of fibroblasts via DR targets. Thus, R5S4TRAIL may serve as a promising candidate for RIPF treatment.

## Data Availability

The original contributions presented in the study are included in the article/**Supplementary Material**. Further inquiries can be directed to the corresponding author.

## Supplementary material

Supplementary Figure 1 body weight changes in different groups.

Supplementary Figure 2 HE staining of main organs in different treatment groups, scale bar=50μm.
