# Supplementary material for: R5S4TRAIL ameliorates radiation-induced pulmonary fibrosis by alleviating inflammatory responses and promoting apoptosis of fibroblasts
Source: Front Immunol. 2025 Jul 31;16:1600776. doi: 10.3389/fimmu.2025.1600776 (PMC12350115; doi:10.3389/fimmu.2025.1600776)
Supplement: Supplementary Figure 1 — body weight changes in different groups. [file SupplementaryFile1.docx]

**
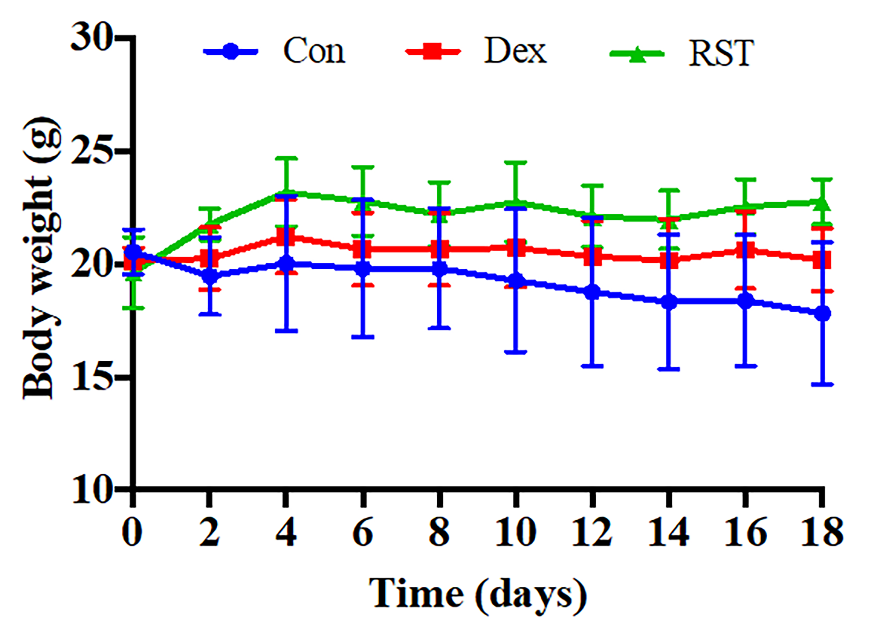
**

**Supplementary figure 1. body weight changes in** **different groups.**

**
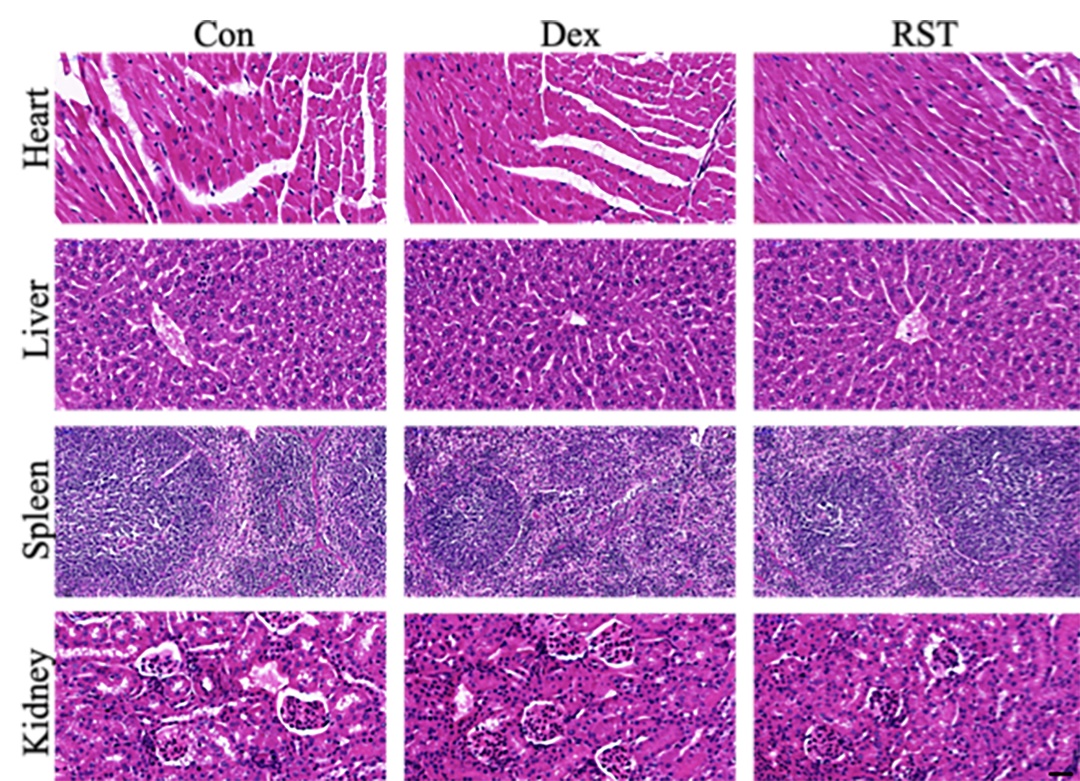
**

**Supplementary figure 2. HE staining for main organs of different treatment groups, scale bar=50μm**
